# Prevalence Rates of Mental Disorders in Chilean Prisons

**DOI:** 10.1371/journal.pone.0069109

**Published:** 2013-07-22

**Authors:** Adrian P. Mundt, Rubén Alvarado, Rosemarie Fritsch, Catalina Poblete, Carolina Villagra, Sinja Kastner, Stefan Priebe

**Affiliations:** 1 Department of Psychiatry and Mental Health, Hospital Clínico Universidad de Chile, Santiago, Chile; 2 Unit for Social & Community Psychiatry, Barts & The London School of Medicine & Dentistry, Queen Mary, University of London, London, United Kingdom; 3 School of Public Health, Faculty of Medicine, Universidad de Chile, Santiago, Chile; 4 Department of Psychiatry, Faculty of Medicine, Universidad de los Andes, Santiago, Chile; 5 Department of Child- and Adolescent Psychiatry, Universidad de Chile, Santiago, Chile; 6 Department of Psychology, Universidad de Chile, Santiago, Chile; 7 Department of Psychiatry and Psychotherapy, Charité Campus Mitte, Universitätsmedizin Berlin, Berlin, Germany; Catholic University of Sacred Heart of Rome, Italy

## Abstract

**Objective:**

High rates of mental disorders have been reported for prison populations worldwide, particularly in low- and middle-income countries (LMICs). The present study aimed to establish prevalence rates of mental disorders in Chilean prisoners.

**Method:**

A nationwide random sample of 1008 prisoners was assessed in 7 penal institutions throughout Chile. Twelve-month prevalence rates were established using the Composite International Diagnostic Interview (CIDI) and compared to the prevalence rates previously published for the general population.

**Results:**

Prevalence rates were 12.2% (95% CI, 10.2-14.1) for any substance use disorder, 8.3% (6.6-10.0) for anxiety disorders, 8.1% (6.5-9.8) for affective disorders, 5.7% (4.4-7.1) for intermittent explosive disorders, 2.2% (1.4-3.2) for ADHD of the adult, and 0.8% (0.3-1.3) for non-affective psychoses. Significantly higher prevalence rates among prisoners as compared to the general population in Chile were seen for major depression (6.1% vs. 3.7% males, Z=2.58, p<0.05) and illicit drug use (3.3% vs. 0.6% males with drug abuse, Z=2.04, p<0.05; 2.6% vs. 0.1% females with drug abuse, Z=5.36, p<0.001; 3.4% vs. 1.1% males with drug dependence, Z=3.70; p<0.001). Dysthymia (6.5% vs. 15.6%, Z=-2.39, p<0.05), simple (3.3% vs. 11.5%, Z=-3.13, p<0.001) and social phobias (3.9% vs. 9.7%, Z=2.38, p<0.05) were significantly less frequent in the female prison population than in the general population. One-year prevalence rates of alcohol abuse (2.3% vs. 3.9%; Z=-2.04; p<0.05) and dependence (2.7% vs. 8.2%; Z=-5.24; p<0.001) were less prevalent in the male prison population than in the general population.

**Conclusions:**

Service provision for prison populations in Chile should acknowledge high rates of depression and illicit drug use. Overall prevalence rates are lower than reported in other LMICs. Previous research in prison populations in LMICs might have overestimated prevalence rates of mental disorders.

## Introduction

There are about 10 million people worldwide in prisons at a time [1]. The world prison population is growing by about one million per decade [1]. In recent years, there has been growing interest in the mental health of prison populations [2,3]. High rates of mental disorders facing low treatment capacities have given rise to concern. In several countries, special diversion programmes were initiated to keep people with mental disorders from being incarcerated [4]. They serve to reduce the period of imprisonment of people with severe mental disorders [5]. It is not yet clear whether high rates of people with mental disorders in prisons are linked to reduced capacities of institutionalized mental health care [6,7]. Criminal offenders with mental disorders who do not undergo adequate treatment may enter in a cycle of recidivism regarding both the mental disorders and the criminal offenses [8,9]. Imprisonment may constitute an opportunity to reach medically underserved populations [10]. Services in prisons in Western high-income countries are still not equivalent to those in the community and reports suggest that they do not yet meet the mental health needs of the prisoners [11]. Neglected health needs of prison inmates give rise to human rights concerns [12].

There has been a debate as to whether mental disorders are on the rise within prison populations worldwide [2]. Systematic reviews suggest that mental disorders are more prevalent in prison populations than in the general population of the same country [2]. Pooled prevalence rates of psychosis were estimated to be 3.6% (95% CI 3.1-4.2) in male and 3.9% (95% CI 2.7-5.0) in female prisoners. The prevalence rates of major depression were estimated to be 10.2% (95% CI 8.8-11.7) in male and 14.1% (95% CI 10.2-18.1) in female prisoners worldwide. However, prevalence rates of prison populations and the general population have not yet been systematically compared within the same country. The majority of the world prison population lives in low- and middle-income countries (LMICs) [1]. Prevalence rates of mental disorders have only been reported from 7 LMICs: Brazil [13], India [14], Iran [15], Malaysia [16], Mexico [17], Nigeria [18,19] and South Africa [20]. In the only review that has reported aggregated data from LMICs, Kuwait and Dubai were also added to a group of LMICs [2]. LMICs were estimated to have higher prevalence rates of psychosis 5.5% (95% CI 4.2-6.8; p=0.035) than high-income countries. However, some of the 7 studies from LMICs have methodological shortcomings and some were conducted in selective and small samples. Five of the 7 studies recruited the samples from one single penal institution, one from two and one from three penal institutions. Since the conditions vary between the institutions, they may not be representative for the penitentiary system of a country. One may conclude, that the findings from those 7 studies may still be preliminary and possibly inaccurate. Further methodologically sound studies especially from LMICs are needed [2]. Penal institutions in LMICs tend to be so poorly resourced that people with mental disorders are at a particular risk of abuse and human rights violations. It is therefore a question of ethical importance and public health priority to study the type and frequency of mental disorders in prison populations in LMICs so that calls for a better allocation of resources for mental health care in prisons can be underpinned with reliable data. The aim of the present study was to establish prevalence rates of mental disorders in the prison system in Chile and compare prevalence rates in prison populations with those in the general population [21].

## Methods

### Ethics statement

The study was conducted in accordance with the Declaration of Helsinki on ethical principles for medical research involving human subjects. The Ministry of Justice of the Republic of Chile approved of the study. The ethics committee and institutional review board of the University of Chile approved of the study (reference number 342/12). We used a standard written informed consent procedure approved by the ethics committee of the University of Chile. The inclusion of subjects was voluntary upon written informed consent. The capacity of giving informed consent was inferred from the capacity to understand the information about the study and explain the goals back to the interviewer. Age 15 or older and the capacity to give written informed consent were inclusion criteria. Minors of 15 to 17 years were subjected to the same informed consent procedure as adults of 18 years and older. Relatives or guardians were not involved in the written informed consent procedure. All potential participants who declined or otherwise did not participate were eligible for treatment as usual and were not disadvantaged in any other way by not participating in the study.

### Design

We conducted a cross-sectional survey among 1008 of the 46,825 prisoners in closed correctional facilities in Chile. The sample was recruited from 7 penal institutions located in 6 different cities and 5 regions of the country. The sample was stratified for the 5 regions of the country (north, north-central, central, south-central, south), proportionately to the size of the prison population in each region.

### Procedures

The participants were randomly selected from the 7 penal institutions throughout Chile (located in Arica, Valparaíso, 2 in Santiago, Rancagua, Concepción and Temuco). High security sectors within the prisons for inmates showing uncooperative, violent behaviours during detention or high numbers of daily insurgencies were excluded for safety reasons. Sectors of some of the penal institutions were excluded from the sampling due to the lack of basic infrastructure such as a separate room to conduct the interviews. Privacy of the information was assured to all possible participants. All interviewees provided written informed consent. The refusal rate was 1.0%. Two medical doctors and one information technologist were trained in using the Composite International Diagnostic Interview (CIDI) in the Institute of Psychiatry Ramón de Fuentes in Mexico, a WHO accredited centre. The team trained 28 interviewers in Chile for the fieldwork. The 28 trained fieldworkers conducted the interviews between August and October 2007. The field workers were selected on the basis of being professionals with complete tertiary level education related to the topic and setting of the study. They had different professions such as social scientists, sociologists, psychologists and social workers. All of the field workers were trained in the use of the CIDI in a two-day workshop with supervised practice interviews, role-plays and discussions of each question. The trainers evaluated the interviewers’ understanding of the purpose of the study, the purpose of the instrument and of the content of each question. Internal quality control was assured through retraining and mentoring in the field. Unannounced field visits supervising single interviews were carried out. Weekly scheduled meetings with the interviewers and field executives were held for problem identification and troubleshooting.

### Instruments

The interview schedule included socio-demographic characteristics and a computerized version of the CIDI 3.0, a fully structured lay administered interview to establish DSM-IV diagnoses [22]. The instrument has a satisfactory concordance with professionally administered interview schedules [23]. It has been shown that non-clinicians can reliably use the CIDI after a relatively short training period [24]. The CIDI has shown good to excellent Kappa coefficients in test–retest and interrater reliability studies [24]. We opted for the 12-month prevalence rates, rather than 30-day or lifetime prevalence rates for its relevance regarding the need of treatment.

For the comparison of the prison and the general population, we compared prevalence rates separately for males and females. Data from the largest (n=2978), representative population based survey in Chile conducted with the CIDI (versions 1.0 and 1.1) in the 1990s was used for this comparison [21]. Both age adapted and non-adapted comparisons were made.

### Analysis

Prevalence rates were calculated as per cent values with 95% confidence intervals with SPSS® statistical software version 19. For the comparison of frequencies a two-sample calculator for proportions with Stata® statistical software version 12.0 was used. Z scores > ±1.96 and p values <0.05 were considered statistically significant corresponding to the significance levels of the two-sided test. 

## Results

### Socio-demographic characteristics

The final sample included 1008 inmates from five different regions in the country (Table 1). The mean age of the sample was 32.6 ± 10.1 years; 84.8% were male and 15.2% female. There was information on the legal status of 877 subjects, of which a majority of 79.6% were convicts and 20.4% pre-trial or under trial detainees. About one fourth of the sample (25.8%) had a total time in detention of less than 12 months. The mean time spent in detention was 66.7 months. The mean number of people per cell was 5.1 for the included subjects (Table 1).

**Table 1:** socio-demographic characteristics of the sample.

Sample size		n=1008
Gender		
	Male	84.8% (n=855)
	Female	15.2% (n=153)
Age	Mean	32.6 ± 10.1 years
	15-24 years	24.8% (n=250)
	25-34 years	36.9% (n=372)
	35-44 years	25.5% (n=257)
	45-54 years	9.4% (n=95)
	55-64 years	2.9% (n=29)
	>65 years	0.5% (n=5)
Legal status (n=877)		
	Convicts	79.6% (n=698)
	Pre-trial detainees	20.4% (n=179)
Total time in detention (n=990)	Mean	58.4 (95% CI; 54.7-62.1)
≤12 months total time in detention (n=990)		25.8% (n=255)
>12 months total time in detention (n=990)		74.2% (n=735)
Number of people sharing the same cell	Mean	5.1 (95% CI; 4.7-5.7)
Location		
	North (Arica)	15.2% (n=153)
	North-central (Valparaíso)	11.2% (n=116)
	Central (Santiago)	36.9% (n=372)
	South-central (Rancagua, Concepción)	22.9% (n=231)
	South (Temuco)	13.5% (n=136)

### Prevalence rates of mental disorders in Chilean prisons

The 12-month prevalence rate for any mental disorder was 26.6% (95% CI, 24.0-29.3). The prevalence rate for any substance use disorder was 12.2% (95% CI, 10.2-14.1); within that group 6.6% (95% CI, 5.2-8.1) of the total sample had disorders associated with illicit drug use. The 12-month prevalence rates for the group of anxiety disorders was 8.3% (95% CI, 6.6-10.0), for the group of affective disorders 8.1% (95% CI, 6.5-9.8); within that group 6.9% (95% CI, 5.4-8.6) had major depressive disorders (Table 2).

**Table 2:** Prevalence rates of mental disorders in Chilean prison populations.

**Group**	**Disorder**	**Phenomenon**	**Prevalence rate**	**95% Confidence interval**
Any mental disorder			26.6%	(24.0-29.3)
Substance use disorder			12.2%	(10.2-14.1)
	Illicit drug use disorder		6.6%	(5.2-8.1)
		Abuse	3.2%	(2.1-4.4)
		Dependence	3.5%	(2.4-4.7)
	Alcohol use disorder		4.7%	(3.4-5.9)
		Abuse	2.1%	(1.2-3.0)
		Dependence	2.6%	(1.6-3.6)
	Nicotine use disorder	Dependence	4.4%	(3.2-5.8)
Affective disorders			8.1%	(6.5-9.8)
	Major depression		6.9%	(5.4-8.6)
	Minor depression		0.4%	(0.1-0.8)
	Dysthymia		1.1%	(0.5-1.8)
	Mania		1.3%	(0.7-2.0)
Anxiety disorders			8.3%	(6.6-10.0)
	Generalised anxiety disorder		0.8%	(0.3-1.4)
	Simple phobia		3.1%	(2.0-4.2)
		Agoraphobia	0.4%	(0.1-0.8)
	Social phobia		3.2%	(2.3-4.4)
	Panic disorder		1.2%	(0.6-1.9)
	Adult separation anxiety disorder		2.5%	(1.6-3.5)
	PTSD		1.1%	(0.5-1.8)
	Obsessive compulsive disorder		0.2%	
Other disorders			7.2%	(5.7-8.9)
	ADHD of the adult		2.2%	(1.4-3.2)
	Intermittent explosive disorder		5.7%	(4.4-7.1)
	Anorexia		0	
	Bulimia		0.1%	
	Pathological gambling		0	
Possible non-affective psychosis			0.8%	(0.3-1.3)

### Differences of prevalence rates for short and long term prisoners

Subjects with a detention time ≤12 months had a higher rate (35.7%) for any mental disorder than subjects with >12 months in detention (23.1%; p<0.001; Table 3). Alcohol use disorders were significantly more common (12.6%; 95% CI 8.5-16.7) in inmates with a detention time ≤12 months as compared to inmates with >12 months in detention (2.1%; 95% CI 1.1-3.1) and illicit drug use disorders were more common for the prisoners ≤12 months in detention (8.5%; 95% CI 5.1-11.9) as compared to the ones >12 months in detention (3.5%; 95% CI 2.2-4.8). Since the reported data are 12-month prevalence rates the latter numbers correspond to the ones reporting continuous substance use during imprisonment. Nicotine dependence was also significantly more common among those with a detention time of less than 12 months. There was no statistically significant difference for any of the other disorders. Depression was more frequent in the ones with lower detention time, mania more frequent among the ones with longer detention time, but the differences were not statistically significant.

**Table 3:** Prevalence rates of prisoners with ≤12 and >12 months of time in detention.

	**Prison Population (n=990)**				
	**≤12 months in detention (n=255)**		**>12 months in detention (n=735)**		**Two-sample z-test of proportions**
Disorder	Prevalence rate	95% CI	Prevalence rate	95% CI	
Any disorder	**35.7%**	(29.8-42.0)	23.1%	(20.1-26.0)	Z=3.94^**^
Any affective disorder	9.0%	(5.5-12.9)	7.6%	(5.7-9.5)	Z=0.71^n.s.^
Major depressive episode	8.2%	(5.1-12.1)	6.4%	(4.6-8.2)	Z=0.98^n.s.^
Dysthymia	0.4%	(0.0-1.2)	1.2%	(0.5-2.0)	Z=-1.11^n.s.^
Mania	0.0%		1.4%	(0.5-2.2)	Z=-1.68^n.s.^
Anxiety disorder	8.2%	(5.1-11.8)	8.3%	(6.4-10.2)	Z=-0.05^n.s.^
Generalized anxiety disorder	1.2%	(0.0-2.7)	0.7%	(0.1-1.2)	Z=0.76^n.s.^
Simple phobia	3.9%	(1.6-6.7)	2.9%	(1.8-4.1)	Z=0.97^n.s.^
Agoraphobia	0.0		0.5%	(0.1-1.1)	Z=-0.71^n.s.^
Social phobia	3.1%	(1.2-5.5)	3.0%	(1.8-4.4)	Z=0.08^n.s.^
Panic disorder	0.4%	(0.0-1.2)	1.5%	(0.7-2.4)	Z=-1.29^n.s.^
Post-traumatic stress disorder	0.4%	(0.4-1.2)	1.4%	(0.7-2.2)	Z=-0.94^n.s.^
Any substance use disorder	**22.7%**	(17.6-28.2)	8.7%	(6.7-11.0)	Z=5.86^**^
Alcohol abuse	**6.3%**	(3.5-9.4)	0.7%	(0.1-1.4)	Z=5.32^**^
Alcohol dependence	**6.3%**	(3.5-9.4)	1.4%	(0.5-2.2)	Z=4.19^**^
Illicit drug abuse	**5.1%**	(2.4-7.8)	2.4%	(1.5-3.5)	Z=2.14^*^
Illicit drug dependence	**3.4%**	(2.3-4.8)	1.1%	(0.6-1.8)	Z=2.45^*^
Nicotine dependence	**8.2%**	(5.1-11.8)	1.9%	(1.0-3.0)	Z=4.70^**^
Possible non-affective psychosis	1.2%	(0.7-2.7)	0.5%	(0.0-1.1)	Z=1.17^n.s.^
Intermittent explosive disorder	7.1%	(3.9-10.2)	5.0%	(3.5-6.7)	Z=1.26^n.s.^
Attention deficit disorder	2.4%	(0.8-4.3)	2.2%	(1.1-3.3)	Z=0.19^n.s.^

^n s^. not significant

* p<0.05

** p<0.001

### Comparison of prevalence rates in the Chilean prison population and general population

There were different patterns of mental disorders in the prison population and the general population (Table 4): Significantly higher prevalence rates among prisoners as compared to the general population in Chile were seen for major depression (6.1% vs. 3.7% males, Z=2.58, p<0.05) and illicit drug use (3.3% vs. 0.6% males with drug abuse, Z=2.04, p<0.05; 2.6% vs. 0.1% females with drug abuse, Z=5.36, p<0.001; 3.4% vs. 1.1% males with drug dependence, Z=3.70; p<0.001). To the contrary, dysthymia (6.5% vs. 15.6%, Z=-2.39, p<0.05), simple (3.3% vs. 11.5%, Z=-3.13, p<0.001) and social phobias (3.9% vs. 9.7%, Z=2.38, p<0.05) were significantly less frequent in the female prison populations than in the general population. One-year prevalence of alcohol abuse (2.3% vs. 3.9%; Z=-2.04; p<0.05) and dependence (2.7% vs. 8.2%; Z=-5.24; p<0.001) were less prevalent in the male prison population than in the general population. There was a trend for higher rates of non-affective psychosis in the prison population (p<0.1 for the two-sided z-test; if presuming higher rates in the prison population and performing the one-sided z-test, significance levels of <0.05 would have been reached). When the data was weighted for age, the differences remained significant for the same categories.

**Table 4:** Comparison of the prevalence rates between the prison population and the general population, presented separately for males and females.

	**Male**					**Female**				
	**Prison population (n=855)**		**General population**[21]		**Two-sample z-test of proportions**	**Prison population (n=153)**		**General population**[21]		**Two-sample z-test of proportions**
**Disorder**	Prevalence rate	95% CI	Prevalence rate	95% CI		Prevalence rate	95% CI	Prevalence rate	95% CI	
Any affective disorder	7.6%	(5.9-9.6)	5.7%	(4.5-7.1)	Z=1.75^n.s.^	11.1%	(6.6-17.2)	12.6%	(11.1-14.3)	Z=-0.54^n.s.^
Major depressive episode	**6.1%**	(4.9-7.9)	3.7%	(2.7-4.8)	Z=2.58^*^	11.1%	(6.6-17.2)	7.5%	(6.3-8.8)	Z=1.59^n.s.^
Dysthymia	1.1%	(0.5-2.2)	1.6%	(1.0-2.5)	Z=-0.96^n.s.^	1.3%	(0.2-4.6)	**5.9%**	(4.8-7.1)	Z=-2.39^*^
Mania	1.4%	(0.7-2.4)	0.7%	(0.3-1.3)	Z=1.61^n.s.^	0.7%	(0.0-3.6)	2.1%	(1.5-2.9)	Z=-1.19^n.s.^
Anxiety disorder^a^	4.3%	(3.5-5.7)	3.7%	(2.7-4.8)	Z=0.70^n.s.^	6.5%	(3.3-10.5)	**15.6%**	(14.0-17.6)	Z=-3.03^*^
Generalized anxiety disorder	0.9%	(0.4-1.8)	0.7%	(0.3-1.3)	Z=0.51^n.s.^	0.0%		1.1%	(0.4-1.8)	Z=-1.30^n.s.^
Simple phobia	3.0%	(2.0-4.4)	3.8%	(2.8-5.0)	Z=-0.99^n.s.^	3.3%	(3.1-7.5)	**11.5%**	(10.0-13.1)	Z=-3.13^**^
Social phobia	3.0%	(2.0-4.4)	2.5%	(1.7-3.5)	Z=0.70^n.s.^	3.9%	(1.5-8.3)	**9.7%**	(8.4-11.2)	Z=-2.38^*^
Obsessive-compulsive disorder	0.2%	(0.0-0.5)	0.7%	(0.2-1.2)	Z=-1.6^n.s.^	0.0%		1.6%	(1.0-2.2)	Z=-1.58^n.s.^
Post-traumatic stress disorder	0.7%	(0.3-1.5)	1.1%	(0.6-1.8)	Z=-0.94^n.s.^	3.3%	(3.1-7.5)	2.4%	(1.7-3.3)	Z=0.69^n.s.^
Any substance use disorder	12.9%	(10.9-15.2)	14.4%	(12.5-16.3)	Z=-0.98^n.s.^	8.5%	(4.6-13.1)	6.7%	(5.5-7.9)	Z=0.84^n.s.^
Alcohol abuse	2.3%	(1.4-3.4)	**3.9%**	(2.9-5.1)	Z=-2.04^*^	0.7%	(0.0-2.0)	0.8%	(0.5-1.4)	Z=-0.13^n.s.^
Alcohol dependence	2.7%	(1.7-4.0)	**8.2%**	(6.8-9.8)	Z=-5.24^**^	2.0%	(0.4-5.6)	1.4%	(0.9-2.1)	Z=0.59^n.s.^
Illicit drug abuse	**3.3%**	(2.1-4.6)	0.6%	(0.3-1.2)	Z=2.04^*^	**2.6%**	(0.7-5.2)	0.1%	(0.0-0.4)	Z=5.36^**^
Illicit drug dependence	**3.4%**	(2.3-4.8)	1.1%	(0.6-1.8)	Z=3.70^**^	3.9%	(1.5-8.3)	2.0%	(1.4-2.8)	Z=1.55^n.s.^
Nicotine dependence	**4.9%**	(3.5-6.3)	2.9%	(2.0-3.8)	Z=2.39^*^	1.3%	(0.0-3.3)	3.1%	(2.3-3.9)	Z=-1.26^n.s.^
Possible non-affective psychosis	0.7%	(0.3-1.5)	0.2%	(0.0-0.7)	Z=1.79^n.s.^	1.3%	(0.2-4.6)	1.1%	(0.7-1.7)	Z=0.23^n.s.^

a anxiety disorders without social phobia and obsessive-compulsive disorders

^n s^. not significant

* p<0.05

** p<0.001

### Co-morbidity

Co-morbidity rates are reported for the two most important diagnostic categories, major depression and illicit drug use disorders. Most frequent co-morbid diagnoses in people with illicit drug use (n= 70) were anxiety disorders and intermittent explosive disorder with 16.7% (95% CI 7.7-25.8) each. Affective disorders were co-morbid in 12.1% (95% CI 4.3-19.9) and ADHD in 10.6% (95% CI 3.2-18.0) of the people with illicit drug use. People with major depressive disorders (n= 67) had most commonly comorbid anxiety disorder (43.5%; 95% CI 31.9-55.8), substance use disorders (22.8%; 95% CI 13.3-33.1), intermittent explosive disorder (18.9%; 95% CI 9.7-28.1) and ADHD (7.2%; 95% CI 1.1-13.3).

## Discussion

As compared to the general population, prisoners in Chile had higher prevalence rates for illicit drug use disorders and major depression. Anxiety disorders in female prisoners and alcohol use disorders in male prisoners were less frequent than in the general population. Prisoners with less than one-year detention time had higher one-year prevalence rates of substance use disorders than prisoners with more than one year in detention. Regarding the co-occurrence of mental disorders, major depression had high rates of co-morbidity. Overall, the prevalence rates for mental disorders identified in Chilean prisons in this study are lower than the estimates for other low- and middle-income countries [2].

### Strengths and limitations

This is the largest and most systematic prevalence study of mental disorders in prison populations from a non-Western middle-income country using a standardized structured interview to generate diagnoses. It is the first study that compares nationwide data from prison populations with nationwide data from a population based study, which both used the same standardized interview schedule. A limitation for the comparison of the prevalence data between the prison population and the general population arises from a time lag of 8-15 years between the two studies. The prevalence data in the general population might have changed over time.

As in most prison mental health studies, there were specific challenges regarding the sampling. We had to exclude high security sectors and prisoners with violent or uncooperative behaviours to assure the safety of the fieldworkers. We also excluded sectors within the prisons that were so poorly resourced that they could not provide the infrastructure required to implement the study such as an interview room. Even though treatment capacities for prison inmates were still extremely low at the time of the study, we cannot exclude that few subjects were successfully treated because we did not collect information on current medication or treatment.

### Comparison against the literature

There are several limitations for the comparison with studies from other countries: 1. The sampling, as explained above, may have been selective for prisoners with cooperative behaviours. It can be assumed most prison mental health studies are limited in reaching prisoners with violent, dangerous or uncooperative behaviours. 2. Other studies may refer to lifetime or current prevalence rates rather than the 12-month prevalence rates used in this study. 3. Different studies applied different structured psychometric instruments to generate the diagnoses. An alternative to the CIDI used in our study is the Structured Clinical Interview for DSM-axis I and II (SCID I and II) used by other groups for studying prevalence rates in prison populations [25]. The SCID has the advantage over the CIDI that it includes diagnoses of several personality disorders if both parts are applied. It may produce a higher level of diagnostic accuracy, because it is professionally administered. At the same time, this is a disadvantage for poorly resourced settings. Several recently published prison mental health studies used the Mini International Neuropsychiatric Interview [13,20,26], which is similarly to the CIDI a fully structured lay administered interview schedule. It has the advantage that its use is easier to train and teach, it is quicker to administer and it has a section for the detection of antisocial personality disorder, which is relevant for prison populations [27]. For international comparisons of prevalence data, consistent instruments and equivalent time spans should be used (e.g. point prevalence, one-year prevalence or life-time prevalence).

Despite these limitations and the heterogeneous findings of the previous 7 studies from LMICs, our results should be seen against the background of other studies: In comparison, the prevalence rates of mental disorders reported here are lower [13,15]. A study in the closed correctional system of Brazil reported rates of 17.6% for lifetime depression, 5.2% for current depression, 6.6% for lifetime bipolar disorder, 26.6% for lifetime alcohol addiction, 27.9% for lifetime drug addiction, and 1.4% for psychotic disorders [13]. Data reported from India were part of an intervention study including mainly pre-trial detainees. Prior to the intervention, prisoners in two jails were screened for severe mental disorders (psychosis and mood disorders) and a 10% prevalence rate was found [14]. A study from Iran reported 3.1% current and 3.9% lifetime prevalence for psychotic disorders, 30.6% current and 48.7% lifetime prevalence for mood disorders, 11.2% current and 78.0% lifetime prevalence for substance use disorders. Different low- and middle-income countries present different legal and cultural frameworks. Iran employs, as other Middle-Eastern countries, strict laws on substance use, which may explain in part that very high rates of substance use problems were found in the prison population. Rates of current psychotic and mood disorders were also higher than the 12-month prevalence rates found in our study [15]. A prison mental health survey conducted in Malaysia primarily targeted HIV positive prisoners and the comorbidity of HIV, mental and substance use disorders [16]. A study from Mexico on mental disorders of female prisoners showed a prevalence rate of 62% for depression in a selective sample with a history of a substance use disorder [17]. The study from Nigeria in 100 inmates reported a lifetime prevalence of 25% for substance use disorders. Two cases of schizophrenia, two cases of major depression and twenty-one cases of recurrent mild depression were in the sample [18]. The most recently published prison mental health survey from South Africa reported that 42% had a substance use disorder, 10.4% current major depression, 24.9% lifetime depression, 4.7% current psychosis and 7.3% lifetime prevalence of psychosis [20]. Especially current non-affective psychotic episodes may have been overestimated by previous research. Apart from a general difficulty to some diagnoses, especially current active schizophrenia, with diagnostic structured interviews, the different interview schedules may have different thresholds for producing positive scores [28].

Even as compared to high-income countries the prevalence rates found in our study appear to be low [2]. In a sample of long-term prisoners in Sweden, 40% of the participants were estimated to have ADHD as compared to 2.2% in our sample [29]. There are several possible explanations for the moderate prevalence rates: 1. Chile has 305 prisoners per 100,000 population, one of the highest prison population rates in Latin America [1], more than double of the worldwide and South American averages. As for many other countries, the prison population in Chile has more than doubled since 1992 [30]. When prison population rates are very high, the prison population may be more similar to the general population regarding their mental health than in countries with lower prison population rates.

2. There was a majority of long term convicts in our sample who show lower rates especially of substance use disorders than recently admitted prisoners. Levels of distress may be highest shortly after admission because of adaptation to the adverse conditions of imprisonment over time. Reduced rates of overall mental disorders were found in a longitudinal study of the effects of long-term imprisonment in a Western setting including a stabilization of pathological personality traits [31]. Imprisonment may hinder use of drugs and alcohol, but does not preclude it since these substances are still available. However, the type of abuse may be different in and outside of prisons. Our data indicate that it is more common to maintain illicit drug use than alcohol use. That is in line with reports from prison inmates about illicit drug dependence inside the institution, whereas it is more unusual to maintain alcohol dependence [32]. There may also be a reporting bias in the sense of underreporting of active substance use inside the prison. Our data underline the weight the penal institutions have regarding the institutionalization of people with substance use disorders. This window of opportunity should be used for the care of those disorders. Even though our study was not longitudinal the results give indirect evidence that prison inmates reduce active substance use to a certain degree during imprisonment. This may neither be sustainable beyond the sentence nor a cost effective way to deal with addiction nor humane but it constitutes a reality for many people affected by substance use disorders. Inmates show high rates of relapse and death from overdose after release from prison [33].

There are two possible explanations for the lower rates of simple and social phobia in the female prison population as compared to the general population: 1. Simple and social phobia may be associated with traits that are protective against engaging in criminal behaviour. 2. Imprisonment may constitute a protective environment characterized by monotony, predictability and avoidance of possible trigger situations for simple and social phobia.

## Conclusions

The findings are not consistent with the hypothesis that severe mental disorders are generally more prevalent in prison populations of low- and middle-income countries than in high-income countries [2]. Previous studies might have overestimated the prevalence of severe mental disorders in LMIC prisons. At the same time, the differences with other findings might also be linked to specific aspects of the context in Chile.

Illicit drug use disorders and major depression may be the most urgent mental health problems to consider when scaling up services for prison populations in Chile with markedly elevated rates as compared to the general population and high rates of co-morbidity. Intervention programmes should focus on prisoners with short term sentences since they have higher prevalence rates and provide the chance of initiating treatment.

Qualitative studies may complete the picture on how people with severe non-affective psychosis are dealt with in prisons in Chile and other LMICs. Other studies using different sampling strategies should provide data on mental disorders shortly after admission and of prisoners with short but possibly recurrent sentences. With respect to research in prison populations in general, the results underline that further studies should distinguish between short and long term prisoners since the mental health problems are likely to be different.
